# Effectiveness of fosfomycin trometamol as oral step-down therapy for bacteraemic urinary tract infections due to MDR *Escherichia coli*: a post hoc analysis of the FOREST randomized trial

**DOI:** 10.1093/jac/dkad147

**Published:** 2023-06-01

**Authors:** Jesús Sojo-Dorado, Inmaculada López-Hernández, Alicia Hernández-Torres, Pilar Retamar-Gentil, Esperanza Merino de Lucas, Laura Escolà-Vergé, Elena Bereciartua, Elisa García-Vázquez, Vicente Pintado, Lucía Boix-Palop, Clara Natera-Kindelán, Luisa Sorlí, Nuria Borrell, Concha Amador-Prous, Evelyn Shaw, Alfredo Jover-Saenz, Jose Molina, Rosa M Martínez-Álvarez, Carlos J Dueñas, Jorge Calvo-Montes, María Lecuona, Virginia Pomar, Irene Borreguero, Virginia Palomo-Jiménez, Fernando Docobo-Pérez, Álvaro Pascual, Jesús Rodríguez-Baño

**Affiliations:** Departamentos de Medicina y Microbiología, Unidad Clínica de Enfermedades Infecciosas y Microbiología, Hospital Universitario Virgen Macarena; Instituto de Biomedicina de Sevilla (IBiS)/CSIC; Universidad de Sevilla, Seville, Spain; CIBER de Enfermedades Infecciosas, Instituto de Salud Carlos III, Madrid, Spain; Departamentos de Medicina y Microbiología, Unidad Clínica de Enfermedades Infecciosas y Microbiología, Hospital Universitario Virgen Macarena; Instituto de Biomedicina de Sevilla (IBiS)/CSIC; Universidad de Sevilla, Seville, Spain; CIBER de Enfermedades Infecciosas, Instituto de Salud Carlos III, Madrid, Spain; Servicio de Medicina Interna, Unidad de Enfermedades Infecciosas, Hospital Clínico Universitario Virgen de la Arrixaca, Murcia, Spain; Departamentos de Medicina y Microbiología, Unidad Clínica de Enfermedades Infecciosas y Microbiología, Hospital Universitario Virgen Macarena; Instituto de Biomedicina de Sevilla (IBiS)/CSIC; Universidad de Sevilla, Seville, Spain; CIBER de Enfermedades Infecciosas, Instituto de Salud Carlos III, Madrid, Spain; Unidad de Enfermedades Infecciosas, Hospital General Universitario Dr Balmis, Instituto de Investigación Sanitaria y Biomédica de Alicante (ISABIAL), Alicante, Spain; Departamento de Medicina Clínica, Universidad Miguel Hernández, Elche, Alicante, Spain; CIBER de Enfermedades Infecciosas, Instituto de Salud Carlos III, Madrid, Spain; Servicio de Enfermedades Infecciosas, Hospital Universitario Vall d’Hebrón, Barcelona, Spain; Unidad de Enfermedades Infecciosas, Hospital Universitario Cruces, Instituto de Investigación Biocruces, Baracaldo, Vizcaya, Spain; Servicio de Medicina Interna, Unidad de Enfermedades Infecciosas, Hospital Clínico Universitario Virgen de la Arrixaca, Murcia, Spain; CIBER de Enfermedades Infecciosas, Instituto de Salud Carlos III, Madrid, Spain; Servicio de Enfermedades Infecciosas, Instituto Ramón y Cajal de Investigación Sanitaria (IRYCIS), Hospital Universitario Ramón y Cajal, Madrid, Spain; Servicio de Enfermedades Infecciosas, Hospital Universitari Mútua Terrassa, Terrassa, Barcelona, Spain; CIBER de Enfermedades Infecciosas, Instituto de Salud Carlos III, Madrid, Spain; Unidad de Gestión Clínica de Enfermedades Infecciosas, Hospital Universitario Reina Sofía, Córdoba, Spain; Servicio de Enfermedades Infecciosas, Hospital del Mar, and Grupo de Investigación en Patología Infecciosa y Antibioterapia, Institut Hospital del Mar d'Investigacions Mèdiques (IMIM), Universitat Pompeu Fabra, Barcelona, Spain; Servicio de Microbiología, Hospital Universitario Son Espases, Palma de Mallorca, Spain; Unidad de Enfermedades Infecciosas, Hospital Marina Baixa, Villajoyosa, Alicante, Spain; CIBER de Enfermedades Infecciosas, Instituto de Salud Carlos III, Madrid, Spain; Servei de Malalties Infeccioses, Hospital Universitari de Bellvitge; Epidemiologia de les Infeccions Bacterianes, Patologia Infecciosa i Transplantament, Institut d’Investigació Biomèdica de Bellvitge (IDIBELL), L’Hospitalet de Llobregat, Barcelona, Spain; Unidad Territorial Infección Nosocomial, Hospital Universitari Arnau de Vilanova, Institut de Recerca Biomèdica de Lleida, Lleida, Spain; CIBER de Enfermedades Infecciosas, Instituto de Salud Carlos III, Madrid, Spain; Unidad Clínica de Enfermedades Infecciosas, Microbiología y Parasitología, Hospital Universitario Virgen del Rocío/Instituto de Biomedicina de Sevilla (IBiS)/CSIC/Departamento de Medicina, Universidad de Sevilla, Seville, Spain; Unidad de Enfermedades Infecciosas, Hospital Royo Villanova, Zaragoza, Spain; Unidad de Enfermedades Infecciosas, Hospital Universitario de Burgos, Burgos, Spain; CIBER de Enfermedades Infecciosas, Instituto de Salud Carlos III, Madrid, Spain; Servicio de Microbiología, Hospital Universitario Marqués de Valdecilla—IDIVAL, Santander, Cantabria, Spain; Servicio de Microbiología y Control de la Infección, Hospital Universitario de Canarias, La Laguna, Spain; Unidad de Enfermedades Infecciosas, Servicio de Medicina Interna, Hospital de la Santa Creu i Sant Pau, Barcelona, Spain; Unidad de Investigación Clínica y apoyo a Ensayos Clínicos (CTU), Hospitales Universitarios Virgen del Rocío y Virgen Macarena, Sevilla, Spain; Departamentos de Medicina y Microbiología, Unidad Clínica de Enfermedades Infecciosas y Microbiología, Hospital Universitario Virgen Macarena; Instituto de Biomedicina de Sevilla (IBiS)/CSIC; Universidad de Sevilla, Seville, Spain; Departamentos de Medicina y Microbiología, Unidad Clínica de Enfermedades Infecciosas y Microbiología, Hospital Universitario Virgen Macarena; Instituto de Biomedicina de Sevilla (IBiS)/CSIC; Universidad de Sevilla, Seville, Spain; Departamentos de Medicina y Microbiología, Unidad Clínica de Enfermedades Infecciosas y Microbiología, Hospital Universitario Virgen Macarena; Instituto de Biomedicina de Sevilla (IBiS)/CSIC; Universidad de Sevilla, Seville, Spain; CIBER de Enfermedades Infecciosas, Instituto de Salud Carlos III, Madrid, Spain; Departamentos de Medicina y Microbiología, Unidad Clínica de Enfermedades Infecciosas y Microbiología, Hospital Universitario Virgen Macarena; Instituto de Biomedicina de Sevilla (IBiS)/CSIC; Universidad de Sevilla, Seville, Spain; CIBER de Enfermedades Infecciosas, Instituto de Salud Carlos III, Madrid, Spain

## Abstract

**Background:**

Fosfomycin is a potentially attractive option as step-down therapy for bacteraemic urinary tract infections (BUTI), but available data are scarce. Our objective was to compare the effectiveness and safety of fosfomycin trometamol and other oral drugs as step-down therapy in patients with BUTI due to MDR *Escherichia coli* (MDR-Ec).

**Methods:**

Participants in the FOREST trial (comparing IV fosfomycin with ceftriaxone or meropenem for BUTI caused by MDR-Ec in 22 Spanish hospitals from June 2014 to December 2018) who were stepped-down to oral fosfomycin (3 g q48h) or other drugs were included. The primary endpoint was clinical and microbiological cure (CMC) 5–7 days after finalization of treatment. A multivariate analysis was performed using logistic regression to estimate the association of oral step-down with fosfomycin with CMC adjusted for confounders.

**Results:**

Overall, 61 patients switched to oral fosfomycin trometamol and 47 to other drugs (cefuroxime axetil, 28; amoxicillin/clavulanic acid and trimethoprim/sulfamethoxazole, 7 each; ciprofloxacin, 5) were included. CMC was reached by 48/61 patients (78.7%) treated with fosfomycin trometamol and 38/47 (80.9%) with other drugs (difference, −2.2; 95% CI: −17.5 to 13.1; *P* = 0.38). Subgroup analyses provided similar results. Relapses occurred in 9/61 (15.0%) and 2/47 (4.3%) of patients, respectively (*P* = 0.03). The adjusted OR for CMC was 1.11 (95% CI: 0.42–3.29, *P* = 0.75). No relevant differences in adverse events were seen.

**Conclusions:**

Fosfomycin trometamol might be a reasonable option as step-down therapy in patients with BUTI due to MDR-Ec but the higher rate of relapses would need further assessment.

## Introduction

Bacteraemic urinary tract infections (BUTI) are frequent, with estimated age-adjusted incidence rates of 20–50 episodes per 100 000 person-years.^1,2^ Although antibiotic treatment is usually started IV, data from observational studies suggest that oral step-down treatment with β-lactams, fluoroquinolones and trimethoprim/sulfamethoxazole in patients with bacteraemia caused by Enterobacterales, and specifically by *E. coli*, either with a urinary tract source or not, is associated with similar outcomes as full IV courses.^3–5^ However, resistance to these drugs is frequent among MDR *E. coli* isolates, particularly those producing ESBLs.^6^ Therefore, there is a medical need for information about the effectiveness and safety of alternative drugs as oral step-down in patients with BUTI.

Fosfomycin remains active against a high proportion of MDR *E. coli* isolates^7^ and might be a potential step-down alternative. However, the low plasma levels obtained with fosfomycin trometamol,^8^ an oral form of the drug, raised doubts about its suitability for bacteraemic infections. In spite of this, a recent randomized trial found that fosfomycin trometamol was non-inferior to ciprofloxacin as oral step-down treatment in 97 women with febrile UTI, of which 50% had a bacteraemic infection.^9^ To the best of our knowledge, there are no specific studies of fosfomycin trometamol performed in BUTI due to MDR *E. coli*, and no data are available for BUTI in men.

In the FOREST trial, IV fosfomycin was compared with ceftriaxone or meropenem as initial targeted therapy for BUTI due to MDR *E. coli*.^10^ Because oral step-down was allowed in both arms, we had the opportunity to compare the efficacy and safety of oral fosfomycin trometamol with other ‘standard’ oral drugs.

## Patients and methods

### Study design and participants

This is a post hoc analysis of the FOREST trial (clinicaltrials.gov identifier: NCT02142751), of which main results were previously published.^10^ FOREST was an academic-driven, multicentre, open-label randomized clinical trial comparing the efficacy and safety of IV fosfomycin with ceftriaxone or meropenem (if the isolate was resistant to ceftriaxone) for the targeted treatment of bacteraemic UTI caused by MDR (i.e. resistant to at least one drug from three or more groups) *E. coli* in adult patients. The study was performed in 22 Spanish hospitals from June 2014 to December 2018.

The study protocol allowed switching to oral therapy according to the treating physician criteria, after a minimum of 4 days of IV treatment if the following conditions were fulfilled: clinical improvement, haemodynamic stability, tolerance to oral intake, and isolate susceptible to one of the permitted oral drugs. The permitted drugs were fosfomycin trometamol (3 g q48h) for patients assigned to IV fosfomycin, and cefuroxime axetil (250 mg q12h), ciprofloxacin (500 mg q12h), amoxicillin/clavulanate (500–125 mg q8h), or trimethoprim/sulfamethoxazole (160–800 mg q12h) for patients assigned to IV ceftriaxone or meropenem. In addition, switch to parenteral ertapenem was allowed for patients with ceftriaxone-resistant isolates assigned to meropenem if no oral drugs were available. For this analysis, only patients in whom step-down to an oral drug was performed were included.

The study was approved by the Andalusian Ethics Committee (registry: CCEIBA 0039/14); written informed consent was obtained from all participants. This report followed the STROBE recommendations (Table S1, available as Supplementary data at *JAC* Online).

### Study variables

The primary endpoint was clinical and microbiological cure (CMC) at the test of cure (TOC; 5–7 days after finalization of treatment). Clinical cure was defined as resolution of all new signs and symptoms of infection, and microbiological cure (or eradication) as no isolation of the causative *E. coli* strain in blood cultures from Day 5 or in urine culture at TOC. The primary endpoint was evaluated using ITT criteria (i.e. patients not evaluated for whatever the reason were considered not to have reached CMC) in the modified ITT population, including all randomized patients who received one dose of a study drug. Secondary endpoints included clinical cure, microbiological eradication, mortality, relapse and reinfection. Relapse was defined as reappearance of fever or UTI symptoms with isolation in blood or urine of *E. coli* with two or more band differences in PFGE, or two or more drugs in susceptibility profile if not available for PFGE. Reinfection was defined similarly but with isolation of a different bacterium or *E. coli* not fulfilling the previously mentioned criteria. The definitions for other endpoints were previously published.^10^ Other patients’ variables collected are listed and defined in Table 1. In addition, adverse events (AEs) reported after the oral therapy had been started were also collected. The patients were followed up for 60 days.

**Table 1.** Baseline characteristics of patients with bloodstream infection due to MDR *E. coli* switched to oral fosfomycin trometamol or alternative drugs after receiving IV antibacterial treatment

Characteristic	Fosfomycin (*n* = 61)	Other drugs (*n* = 47)	*P* value^a^
Age in years, median (IQR)	68 (60–79)	73 (61–84)	0.18^b^
Male sex	31 (50.8)	22 (46.8)	0.67
Charlson index, median (IQR)	1 (0–3)	2 (0.5–3)	0.09^c^
Charlson index ≥3	17 (27.9)	13 (27.7)	0.98
Congestive heart failure	6 (9.8)	5 (10.6)	>0.99^c^
Chronic pulmonary disease	10 (16.4)	5 (19.6)	0.39
Chronic liver disease	2 (3.2)	0	0.50^c^
Diabetes mellitus	18 (29.5)	11 (23.4)	0.47
Chronic renal disease	8 (13.1)	9 (19.1)	0.39
Cancer	13 (21.3)	8 (17.0)	0.57
Bladder catheter at enrolment	18 (29.5)	15 (31.9)	0.78
Invasive procedure in the urinary tract in previous month^d^	11 (18.0)	3 (6.4)	0.07
Immunosuppressive drugs	6 (9.8)	3 (3.4)	0.72^c^
Infection acquisition type^e^			
Community-acquired	29 (47.5)	27 (57.4)	0.30
Healthcare-associated	23 (37.7)	11 (23.4)	0.11
Nosocomial	9 (14.8)	9 (19.1)	0.54
Present infection data			
Low-urinary tract symptoms^f^	35 (57.4)	30 (63.8)	0.49
Flank pain/tenderness	23 (37.7)	18 (38.3)	0.95
Severe sepsis at presentation^g^	14 (23.0)	12 (25.5)	0.75
Pitt score, median (IQR)^h^	1 (0–1)	1 (0–2)	0.72
eGFR <60 mL/min/1.73 m^2^ at enrolment	19 (31.6)	18 (38.2)	0.54
Hydronephrosis in echography at enrolment	8 (13.1)	4 (8.5)	0.45
Early clinical response (Days 5–7)^i^	60 (98.4)	44 (93.6)	0.31^c^
Susceptibility of baseline *E. coli*			
Amoxicillin/clavulanic acid	34 (55.7)	25 (53.2)	0.79
Cefuroxime axetil	28 (45.9)	30 (63.8)	0.06
Ciprofloxacin	11 (18.0)	11 (23.4)	0.49
Trimethoprim/sulfamethoxazole	28 (45.9)	18 (38.3)	0.42
ESBL-producing isolate	29 (47.5)	15 (31.9)	0.10
Median days until active treatment (IQR)	1 (0–2)	0 (0–2)	0.84^b^
Median days with IV antibiotic therapy (IQR)	5 (5–6)	5 (4–6)	0.21^b^
Median days with oral antibiotic therapy (IQR)	5 (4–8)	6 (5–8)	0.18^b^
Median days of total antibiotic therapy (IQR)	11 (9–13)	12 (10–13)	0.24^b^

Data are number of patients (percentage) except where specified. eGFR, estimated glomerular filtration rate.

Chi-squared test unless otherwise indicated.

Mann–Whitney *U* test.

Fisher test.

Included open surgery of the urinary tract, nephrostomy, double-J stent catheter placement, cystoscopy, transurethral resection, transrectal prostate biopsy.

According to Friedman’s criteria.^11^

Included dysuria, urinary frequency or urgency, and suprapubic pain.

Defined according to 2001 SCCM/ESICM/ACCP/ATS/SIS International Sepsis Definitions Conference.^12^

According to reference^13^.

Improvement in all new signs and symptoms of infection.

### Microbiological studies

Bacteria identification and susceptibility testing was performed at local microbiology laboratories using standard techniques. The blood isolates were sent to Hospital Universitario Virgen Macarena, where identification and antimicrobial susceptibility were confirmed using MALDI-TOF and microdilution, respectively, according to EUCAST recommendations^14^; specifically, fosfomycin MIC was studied using agar dilution. ESBL genes were characterized by PCR and sequencing.

### Statistical analysis

The primary analysis was the difference risk calculated as the absolute difference with two-sided 95% CI in the proportion of patients reaching the primary and secondary endpoints in patients switched to oral therapy with fosfomycin trometamol and to other drugs. Subgroup analyses (according to sex, age groups, fosfomycin MIC, urinary catheter, and presence of lumbar pain or tenderness) were performed for the primary endpoint. In addition, because patients were not randomized for being stepped-down to oral therapy, a multivariate analysis was performed using logistic regression to estimate the association of oral step-down with fosfomycin with the primary endpoint controlling for exposure variables with a bivariate *P* value <0.20 in the comparison of both groups; gender was also included. Interactions between CMC and gender, age groups and presence of lumbar pain or tenderness were tested. Finally, we explored the relative risk with 95% CI of CMC according to the exposure to some variables among patients treated with fosfomycin trometamol, with calculation of *P* values by chi-squared or Fisher test for categorical exposures, as appropriate, and Mann–Whitney *U* test for continuous variables. The statistical analyses were performed with SPSS Statistics version 26 (IBM Corp.) and R version 3.6.0 (R Project for Statistical Computing).

## Results

Among the 143 patients included in the modified intention-to-treat population of the FOREST trial, 108 (75.5%) were switched to oral drugs and 35 were not (Figure S1). Those switched to oral drugs were 60/70 randomized to IV fosfomycin and 48/73 randomized to ceftriaxone or meropenem (85.7% versus 65.7%, *P* = 0.006); among the 35 not switched to oral drugs, 13 patients (17.8%, all of them initially treated with meropenem) were switched to parenteral ertapenem.

In order to provide information about the generalizabilty of the results, we compared the 108 patients who were switched to oral drugs with the 35 who were not; the data are shown in Table S2. Although limited by the low numbers, use of immunosuppressant drugs was numerically more frequent among patients who were not switched to oral drugs; also, these patients more frequently had isolates resistant to oral antibiotics, and a lower frequency of early clinical response.

For the comparison of oral drugs, we included 61 patients switched to oral fosfomycin trometamol (the 60 randomized to IV fosfomycin plus 1 patient randomized to meropenem who was switched to oral fosfomycin by mistake) and 47 switched to other drugs (28 to cefuroxime axetil, 7 to amoxicillin/clavulanic acid, 7 to trimethoprim/sulfamethoxazole, and 5 to ciprofloxacin).

The features of patients stepped-down to oral drugs are shown in Table 1. Patients switched to fosfomycin trometamol had a numerically lower median Charlson index (but similar proportion of patients with Charlson ≥3), higher proportion of invasive procedures of the urinary tract in the previous month, and their isolates were more frequently susceptible to cefuroxime. There were no differences in the features of the infection being treated, or in the rates of early clinical and microbiological response, which were >90% in both groups. The mean durations of previous IV treatment and of oral treatment were also similar.

The outcomes are shown in Table 2. CMC was reached by 48/61 patients (78.7%; 95% CI: 66.7–87.2) treated with fosfomycin trometamol and 38/47 (80.9%; 95% CI: 67.2–89.8) with other drugs (absolute difference, −2.2; 95% CI: −17.5 to 13.1; *P* = 0.38). In the other drugs group, CMC was 24/28 (85.7%) in patients with cefuroxime, 5/7 (71.4%) with amoxicillin/clavulanic acid, 5/7 (71.4%) with trimethoprim/sulfamethoxazole and 4/5 (80%) with ciprofloxacin. The CMC rate was higher among patients with ceftriaxone-susceptible isolates compared with those with resistant isolates in both treatment groups. The numerically lower rate of CMC in the fosfomycin groups was due to a lower rate of microbiological eradication (78.6%, 48/61 versus 87.2%, 41/47; *P* = 0.12) whereas clinical cure was similar between groups, with a numerically higher cure rate with fosfomycin among patients with ceftriaxone-susceptible isolates (100%, 29/29 versus 93.1%, 27/29; *P* = 0.07). Relapses (but not reinfections) were more frequent with fosfomycin (15.0%, 9/61 versus 4.3%, 2/47; *P* = 0.03). Two patients treated with fosfomycin died during follow-up and after treatment with fosfomycin had been finished, due to cancer progression and to decompensation of chronic heart and renal insufficiencies, respectively.

**Table 2.** Analysis of the primary endpoint (clinical and microbiological cure) and secondary endpoints

Outcomes	Oral fosfomycin	Other oral drugs	Risk difference (2-sided 95% CI)	2-sided *P* value
Clinical and microbiological cure at TOC				
All patients	48/61 (78.7)^a^	38/47 (80.9)^b^	−2.2 (−17.5 to 13.1)	0.38
Patients with ceftriaxone-susceptible isolates	25/29 (86.2)	25/29 (86.2)	0 (−17.7 to 17.7)	0.50
Patients with ceftriaxone-resistant isolates	23/32 (71.9)	13/18 (72.2)	−0.3 (−26.2 to 25.6)	0.49
Clinical cure at TOC				
All patients	57/61 (93.4)	43/47 (91.4)	2.0 (−8.0 to 12.0)	0.34
Patients with ceftriaxone-susceptible isolates	29/29 (100)	27/29 (93.1)	6.9 (−2.5 to 16.2)	0.07
Patients with ceftriaxone-resistant isolates	28/32 (87.5)	16/18 (88.8)	−1.3 (20.0 to 17.4)	0.44
Microbiological eradication at TOC				
All patients	48/61 (78.6)	41/47 (87.2)	−8.6 (−23.1 to 5.9)	0.12
Patients with ceftriaxone-susceptible isolates	25/29 (86.2)	27/29 (93.1)	−6.9 (−22.5 to 8.7)	0.19
Patients with ceftriaxone-resistant isolates	23/32 (71.8)	14/18 (77.7)	−5.9 (−31.2 to 19.4)	0.32
Other endpoints				
Mortality	2/61 (3.3)	0/47 (0)	3.2 (−1.8 to 8.4)	0.10
Relapses	9/61 (15.0)	2/47 (4.3)	10.7 (−0.8 to 22.2)	0.03
Reinfections	4/61 (6.7)	3/47 (6.5)	0.2 (−9.2 to 9.6)	0.48

All endpoints were evaluated using ITT criteria (i.e. lack of assessment was considered as not reaching clinical cure or microbiological eradication), except for relapses and reinfections, for which only detected events were considered. Data are number of patients with the endpoint/total treated (percentage). TOC, test of cure.

Reasons for not reaching clinical and microbiological cure: lack of urine sample for microbiological assessment, 2; clinical assessment at TOC missing, 2; microbiological failure, 9; clinical failure, 3 (these 3 also had a microbiological failure).

Reasons for not reaching clinical and microbiological cure: clinical assessment at TOC missing, 1; microbiological failure, 5; clinical failure, 3.

The estimation of the association of fosfomycin trometamol with clinical and microbiological cure was adjusted for age, gender, Charlson index, invasive procedure of the urinary tract and ceftriaxone susceptibility of the isolate in a regression model; the adjusted ORs for CMC, clinical cure and microbiological cure were 1.11 (95% CI: 0.42–3.29, *P* = 0.75), 1.97 (95% CI: 0.31–12.47, *P* = 0.47) and 0.77 (95% CI: 0.22–2.37, *P* = 0.58), respectively. In order to control for the effect of previous IV treatment, we could not use this variable together with the variable ‘oral drug’ because of collinearity (all patients treated with IV fosfomycin were switched to oral fosfomycin); therefore, we use the variable ‘previous IV treatment’ instead, and included the same potential confounders except ceftriaxone susceptibility of the isolate (also to avoid collinearity). The adjusted OR for CMC with oral fosfomycin previously treated with IV fosfomycin compared with other oral drugs previously treated with IV ceftriaxone was 0.72 (95% CI: 0.20–2.36; *P* = 0.62), and compared with other oral drugs previously treated with IV meropenem, 2.43 (95% CI: 0.66–9.09; *P* = 0.18). In subgroup analysis, the results for the comparison of fosfomycin and other drugs regarding the primary endpoint were similar to the overall group (Table 3).

**Table 3.** Subgroup analyses for clinical and microbiological cure using ITT criteria

Subgroups	Oral fosfomycin	Other oral drugs	*P* value
Sex			
Men	24/31 (77.4)	18/22 (81.8)	0.74
Women	24/30 (80.0)	20/25 (80.0)	>0.99
Age groups			
≤80 years	34/46 (73.9)	24/32 (75.0)	0.91
>80 years	14/15 (93.3)	14/15 (93.3)	0.85
Charlson index			
≤2	33/44 (75.0)	29/34 (85.3)	0.26
>2	15/17 (88.2)	9/13 (69.2)	0.36^a^
Fosfomycin MIC			
MIC ≤1 mg/L	19/26 (73.1)	13/15 (86.7)	0.31
MIC >1 mg/L	22/27 (81.5)	28/24 (75.0)	0.73^a^
Urinary catheter			
No	32/43 (74.4)	25/32 (78.1)	>0.99^a^
Yes	16/18 (88.9)	13/15 (86.7)	0.71
Lumbar pain/tenderness			
Yes	16/23 (69.6)	13/18 (72.2)	<0.99^a^
No	32/38 (84.2)	25/29 (86.2)	0.82

Data are number of patients with the endpoint/total treated (percentage).

*P* values obtained with Fisher test; all other *P* values with chi-squared test.

Among patients treated with fosfomycin trometamol, we explored whether exposure to some key variables was associated with a different risk of CMC. Regarding sex, CMC with fosfomycin was 77.4% (24/31) in men and 80.0% (24/30) in women [relative risk (RR), 0.96; 95% CI: 0.74–1.25; *P* = 0.80). The median (IQR) duration of previous IV therapy was 5 days (5–6) and 6 days (5–6.5) among those achieving and not achieving CMC (*P* = 0.54); and for oral therapy was 5.5 days (4–8) and 5 days (3.5–6.5), respectively (*P* = 0.35). CMC was 86.2% (25/29) and 71.9% (23/32) among those with ceftriaxone-susceptible and -resistant isolates (RR, 1.19; 95% CI: 0.92–1.55; *P* = 0.17). The outcomes according to fosfomycin MIC are shown in Table 4; no trend towards worse outcomes with higher MIC was evident. All relapses occurred in patients whose isolates had MIC 0.25–8 mg/L.

**Table 4.** Outcomes of patients treated with fosfomycin trometamol as step-down according to the MIC of the *E. coli* isolate

MIC (mg/L)	No. of isolates	No. with clinical and microbiological cure (%)	No. with clinical cure (%)	No. with microbiological cure (%)	No. with relapse (%)
0.50	6	5 (83.3)	5 (83.3)	5 (83.3)	0
1	17	12 (70.6)	15 (93.8)	12 (70.6)	4 (25.0)
2	12	10 (83.3)	12 (100)	10 (83.3)	3 (25.0)
4	9	8 (88.9)	9 (100)	8 (88.9)	2 (22.2)
8	7	5 (71.4)	6 (85.7)	5 (71.4)	0
16	—	—	—	—	—
32	2	1 (50)	2 (100)	1 (50)	0
64	1	1 (100)	1 (100)	1 (100)	0
Missing^a^	7	6 (85.7)	6 (85.7)	7 (100)	0

Isolates unavailable for susceptibility testing at central laboratory.

AEs were reported for 17 patients receiving oral fosfomycin and nine other oral drugs (27.8% versus 19.1%, *P* = 0.38). Diarrhoea was reported in three (4.9%) patients with fosfomycin (one due to *Clostridioides difficile*) and two (4.7%) with comparators; also, two patients in the comparator group had vaginitis and one oral mycosis (none with fosfomycin). Overall, eight AEs were considered severe but none were considered as related to the study drugs; four occurred in patients receiving fosfomycin (spontaneous haematoma, cancer progression with severe hypercalcaemia, fever associated with a UTI recurrence, and biliary tract infection) and four in other drug groups (vertebral fracture after syncope, heart failure, anaemia and acute renal failure).

## Discussion

In the FOREST trial, step-down to any oral therapy was used more frequently in patients assigned to IV fosfomycin than in those assigned to IV ceftriaxone or meropenem, which could be explained by differences in reaching the criteria for oral step-down (as seen by differences in reaching early clinical response) and because susceptibility to other oral drugs was less frequent in the latter (particularly for ceftriaxone-resistant isolates), confirming that fosfomycin trometamol is a potential alternative for oral step-down drug in patients with MDR *E. coli* complicated UTI (cUTI). Also, in this post hoc analysis, when patients stepped-down to oral drugs were considered, fosfomycin trometamol was associated with similar CMC rates as other oral drugs including β-lactams, ciprofloxacin and trimethoprim/sulfamethoxazole. Of note, the rate of early clinical response was similar in patients switched to oral fosfomycin or other oral drugs, suggesting that the primary infection was similarly controlled in both groups when patients were stepped-down to the oral drug. Also, the rate of AEs was similar. However, a higher rate of relapses was seen with fosfomycin trometamol.

Fosfomycin trometamol has been traditionally used for uncomplicated cystitis^15^; however, there is a growing interest in its potential usefulness as an oral alternative for cUTI and as oral step-down after IV treatment.^9,16–18^ A theoretical barrier for the oral use of this drug in invasive UTI has been the low plasma levels achieved.^8^ However, data from a mice model of ascending pyelonephritis suggested an unexpectedly good activity with dosing reproducing a similar AUC obtained in humans after a 3 g oral dose^19^; the authors attributed this effect to higher-than-expected kidney concentration of fosfomycin, and increased activity at acidic pH. Interestingly, fosfomycin was similarly effective *in vivo* against susceptible and resistant isolates, which was attributed to the lower MICs of fosfomycin at acidic pH. The number of patients in our study with MIC >8 mg/L (the current EUCAST breakpoint for oral fosfomycin^20^) was too small to draw firm conclusions in this regard, but we could not see any signal of worse outcomes with increasing MIC. Interestingly, the MIC of fosfomycin was not found to be associated with CMC rate in the overall analysis of the FOREST trial.^10^

Regarding clinical studies, we found only two studies comparing fosfomycin trometamol with other oral drugs as step-down regimens in patients with cUTI and fever, pyelonephritis and/or bacteraemia; their data in comparison with this study are shown in Table 5. Wald-Dickler *et al.*^18^ performed a retrospective cohort study in patients with cUTI receiving a discharge prescription with fosfomycin trometamol (*n* = 110) or ertapenem (*n* = 212); however, only 6.4% of those receiving fosfomycin had been bacteraemic (34.7% in the ertapenem group). The infection was caused by *E. coli* in 77% and 53% of patients with fosfomycin and ertapenem, respectively, and most of them were ESBL producers. The adjusted OR for clinical success with fosfomycin was 1.21 (95% CI: 0.68–2.16). Relapse rate was numerically higher with fosfomycin, but without reaching statistical significance (34.5% versus 26.8%; *P* = 0.2). Interestingly, fosfomycin was administered q24h only in 29 (26.3%) patients, whereas in 59 (53.6%) and in 22 (20%), it was administered q48h or q72h, respectively; no outcome differences were found.

**Table 5.** Features of studies comparing fosfomycin trometamol as oral step-down with other drugs in patients with complicated urinary tract infections including fever, pyelonephritis and/or bacteraemia

Study	Design	Infections studied	No. of patients with FOF (no. with bacteraemia)	Frequency of FOF 3 g dose	Days of previous IV therapy in FOF group	Comparator (no. of patients)	Clinical cure rate	Microbiological cure rate	Relapse rate
Wald-Dickler^18^	Retrospective cohort	cUTI including pyelonephritis	110 (7)	3 g q24h: 29 3 g q48h: 59 3 g q72h: 22	0 days: 15 1–3 days: 38 4–5 days: 44 ≥6 days: 13	ETP (212)	FOF: 65.4%^a^ ETP: 64.1%^a^ aOR 1.21 (95% CI: 0.68–2.16)	Not provided	FOF: 34.5% ETP: 26.8%
Ten Doesschate^9^	Randomized trial	Febrile UTI due to *E. coli* in women	48 (25)	3 g q24h	Mean (SD): 3.4 (1.1)	CIP (49)	FOF: 75%^b^ CIP: 65.2%^b^	FOF: 78.4% CIP: 94.3%	FOF: 4.2% CIP: 0
This study	Ad hoc analysis of randomized trial	Bacteraemic UTI due to MDR *E. coli*	61 (61)	3 g q48h	Median (IQR): 5 (5–6)	CXM (28), AMC (7), SXT (7), CIP (5)	FOF: 93.4%^c^ COMP: 91.4%^c^ aOR: 1.97 (95% CI: 0.31–12.47)	FOF: 78.6% COMP: 87.2%	FOF: 15% COMP: 4.3%

AMC, amoxicillin/clavulanic acid; CIP, ciprofloxacin; COMP, comparators; cUTI, complicated urinary tract infection; CXM, cefuroxime axetil; ETP, ertapenem; FOF, fosfomycin trometamol; SXT, trimethoprim/sulfamethoxazole; UTI, urinary tract infection.

Clinical cure defined as resolution of signs and symptoms of infection without relapse at Day 30.

Clinical cure defined as being alive with reduction of all initial local and systemic febrile UTI-related symptoms, without the requirement of additional antibiotic therapy for UTI, at Days 6–10 post-end of treatment.

Clinical cure defined as resolution of all new signs and symptoms of infection, 5–7 days after end of treatment.

The second study was a double-blind randomized trial in women with febrile UTI due to *E. coli* who were stepped-down to either fosfomycin trometamol (3 g q24h) or ciprofloxacin (500 mg q12h).^8^ Of the 94 patients included, 51.6% had bacteraemia; 6.2% had an ESBL-producing isolate. Fosfomycin trometamol met the non-inferiority pre-established criteria for clinical cure at TOC (Table 5). However, microbiological cure was less frequent with fosfomycin and gastrointestinal AEs were more frequent with fosfomycin.

Overall, comparison of our results with these studies is complex because of the differences in inclusion criteria, features of patients and microorganisms (Table 5). Taken together with our study, the available information strongly suggests that step-down to oral fosfomycin in cUTI caused by *E. coli* including pyelonephritis and bacteraemia after 2–5 days of IV therapy in responding patients has a similar efficacy to β-lactams or fluoroquinolones in terms of clinical cure but may be associated with lower microbiological eradication and/or higher relapse rate. Importantly, the best dosing is not well stablished; 3 g q48h would seem enough for reaching clinical cure according to our data and those of Wald-Dickler *et al.*^18^; however, relapses were less frequent in the study by Ten Doesschate *et al.*^9^ using 3 g q24h, probably at the cost of increasing the rate of gastrointestinal AEs.

Some recent studies have shown that uncomplicated bacteraemia due to Enterobacterales may be treated for only 7 days, mostly with β-lactams or fluoroquinolones.^21,22^ In our study, the median previous duration of IV therapy with fosfomycin was 5 days, and therefore it might be considered that most of the durative effect was already achieved, and might explain the lack of any association or trend with fosfomycin MIC. Although it might be the case, there is no information about the efficacy of short treatment with fosfomycin; in addition, many of the patients would not be classified as uncomplicated. Importantly, the mean duration of IV treatment in the study by Ten Doesschate *et al.*^9^ was only 3.4 days.

Limitations of this study include lack of randomization for oral step-down; therefore, we used multivariate analysis to adjust for confounders. Also, the sample size was limited. The comparators included different drugs, although all had been shown to be similarly effective as step-down options.^4,5^ Strengths include multicentre recruitment, close follow-up of patients, and monitoring of quality of data typical of patients included in randomized trials.^11^

In conclusion, fosfomycin trometamol might be useful as step-down therapy in patients with BUTI due to susceptible *E. coli*, and would be an additional alternative for patients with MDR isolates, particularly when no other oral option is available, or as a β-lactam- and fluoroquinolone-sparing agent. However, the higher rate of relapses found in this study would need further assessment in specific randomized trials for step-down therapy, ideally including different dosing strategies of fosfomycin trometamol.

## Supplementary Material

dkad147_Supplementary_Data

## Transparency declarations

P.R.-G. reports receiving honoraria as speaker in lectures from Angelini, Menarini and Shionogi. C.A.-P. reports personal fees from Janssen and Gilead outside the submitted work; J.C.-M. reports personal fees from Astra-Zeneca, non-financial support from Angelini, Pfizer and Gilead, outside the submitted work. V.P. has participated in accredited educational activities sponsored by MSD, Pfizer and Shionogi and has been a consultant for Pfizer, Shionogi and Correvio. C.J.D. received support from Gilead, Janssen Cilag and Pfizer for attending meetings, and was a member of advisory boards for Janssen Cilag and Gilead. M.L. received honoraria for educational events organized by bioMérieux, and support for attending meetings from Wefen, bioMérieux and Pfizer. R.M.M.-A. reports honoraria as speaker and support for attending meetings from Janssen, Gilead, Pfizer and MSD. All other authors report no conflicts.

## Supplementary data

Tables S1 and S2 and Figure S1 are available as Supplementary data at *JAC* Online.
