# Supplementary material for: Effectiveness of fosfomycin trometamol as oral step-down therapy for bacteraemic urinary tract infections due to MDR Escherichia coli: a post hoc analysis of the FOREST randomized trial
Source: J Antimicrob Chemother. 2023 Jun 1;78(7):1658–66. doi: 10.1093/jac/dkad147 (PMC10775153; doi:10.1093/jac/dkad147)
Supplement: dkad147_Supplementary_Data [file dkad147_supplementary_data.docx]

**TITLE: Effectiveness of fosfomycin trometamol as oral step-down therapy for bacteraemic urinary tract infections due to multidrug-resistant *Escherichia coli*: A post-hoc analysis of the FOREST randomised trial.**

**SUPPLEMENTARY MATERIAL**

Figure S1. Flow chart of patients included.


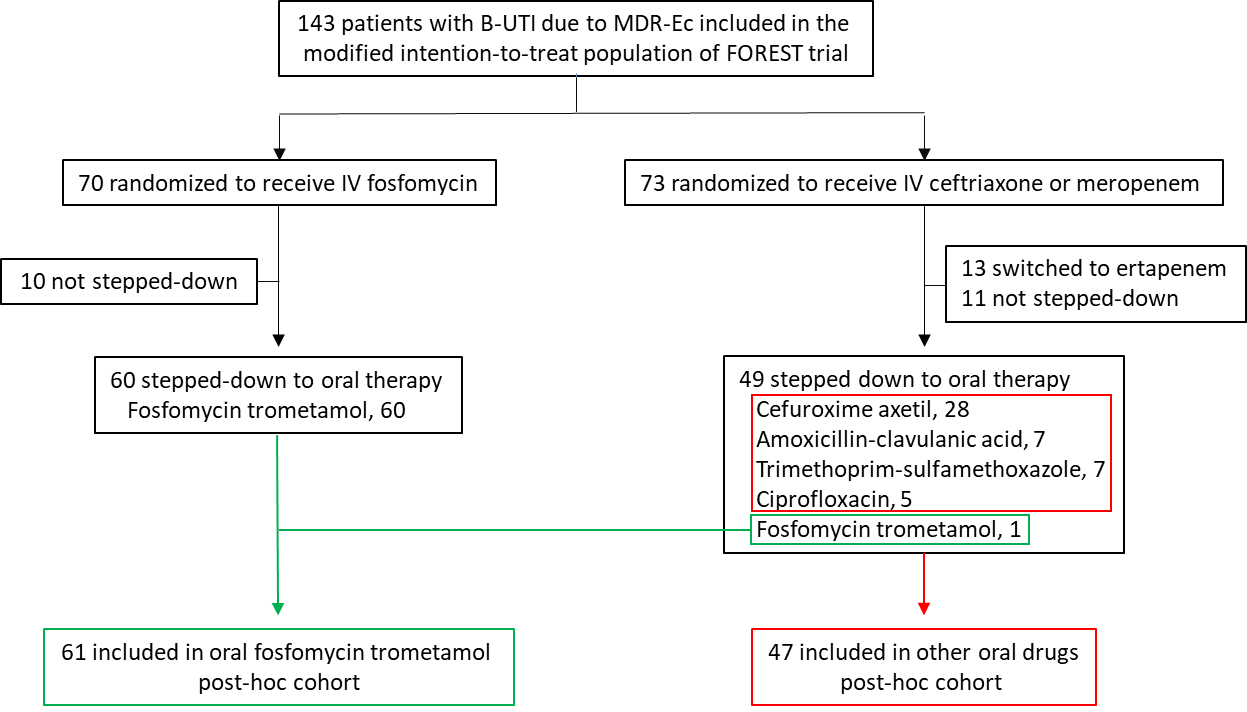


B-UTI: bacteraemic urinary tract infection. MDR-Ec: multidrug-resistant *Escherichia coli*.

Table S1. STROBE checklist.

|  | Item No | | Recommendation | Page no. in manuscript |  |
| --- | --- | --- | --- | --- | --- |
| Title and abstract | 1 | | (a) Indicate the study’s design with a commonly used term in the title or the abstract | 1 |  |
|  |  | | (b) Provide in the abstract an informative and balanced summary of what was done and what was found | 4 |  |
| Introduction | | | |  |  |
| Background/rationale | 2 | | Explain the scientific background and rationale for the investigation being reported | 5 |  |
| Objectives | 3 | | State specific objectives, including any prespecified hypotheses | 5 |  |
| Methods | | | |  |  |
| Study design | 4 | | Present key elements of study design early in the paper | 6 |  |
| Setting | 5 | | Describe the setting, locations, and relevant dates, including periods of recruitment, exposure, follow-up, and data collection | 6 |  |
| Participants | 6 | | (*a*) Give the eligibility criteria, and the sources and methods of selection of participants. Describe methods of follow-up | 6 |  |
|  |  |  | (*b*) For matched studies, give matching criteria and number of exposed and unexposed | NA |  |
| Variables | 7 | | Clearly define all outcomes, exposures, predictors, potential confounders, and effect modifiers. Give diagnostic criteria, if applicable | 7, Table 1 |  |
| Data sources/ measurement | 8* | | For each variable of interest, give sources of data and details of methods of assessment (measurement). Describe comparability of assessment methods if there is more than one group | 7 |  |
| Bias | 9 | | Describe any efforts to address potential sources of bias | 8 |  |
| Study size | 10 | | Explain how the study size was arrived at | NA |  |
| Quantitative variables | 11 | | Explain how quantitative variables were handled in the analyses. If applicable, describe which groupings were chosen and why | NA |  |
| Statistical methods | 12 | | (*a*) Describe all statistical methods, including those used to control for confounding | 8 |  |
|  |  |  | (*b*) Describe any methods used to examine subgroups and interactions | 8 |  |
|  |  |  | (*c*) Explain how missing data were addressed | NA |  |
|  |  |  | (d) Describe any sensitivity analyses | NA |  |
| Results | | | |  | |
| Participants | | 13* | (a) Report numbers of individuals at each stage of study—eg numbers potentially eligible, examined for eligibility, confirmed eligible, included in the study, completing follow-up, and analysed | 8 | |
|  |  |  | (b) Give reasons for non-participation at each stage | Figure S1, Table S1 | |
|  |  |  | (c) Consider use of a flow diagram | Figure S1 | |
| Descriptive data | | 14* | (a) Give characteristics of study participants (eg demographic, clinical, social) and information on exposures and potential confounders | Table 1 | |
|  |  |  | (b) Indicate number of participants with missing data for each variable of interest | NA | |
|  |  |  | (c) Summarise follow-up time (eg, average and total amount) | NA | |
| Outcome data | | 15* | Report numbers of outcome events or summa measures over time | 9 | |
| Main results | | 16 | (*a*) Give unadjusted estimates and, if applicable, confounder-adjusted estimates and their precision (eg, 95% confidence interval). Make clear which confounders were adjusted for and why they were included | 9, Table 2 | |
|  |  |  | (*b*) Report category boundaries when continuous variables were categorized | NA | |
|  |  |  | (*c*) If relevant, consider translating estimates of relative risk into absolute risk for a meaningful time period | NA | |
| Other analyses | | 17 | Report other analyses done—eg analyses of subgroups and interactions, and sensitivity analyses | Tables 3 and 4 | |
| Discussion | | | |  | |
| Key results | | 18 | Summarise key results with reference to study objectives | 11 | |
| Limitations | | 19 | Discuss limitations of the study, taking into account sources of potential bias or imprecision. Discuss both direction and magnitude of any potential bias | 13 | |
| Interpretation | | 20 | Give a cautious overall interpretation of results considering objectives, limitations, multiplicity of analyses, results from similar studies, and other relevant evidence | 11-13 | |
| Generalisability | | 21 | Discuss the generalisability (external validity) of the study results | 11-13 | |
| Other information | | | |  | |
| Funding | | 22 | Give the source of funding and the role of the funders for the present study and, if applicable, for the original study on which the present article is based | 13 | |

Table S2. Features of patients who were switched to oral therapy or not.

| Characteristic | Switch to oral drug  (n=108) | Not switched to oral drug  (n=35)^a^ | P value |
| --- | --- | --- | --- |
| Age in years, median (interquartile range) | 61 (49-71) | 63 (50-75) | 0.30 |
| Male sex | 53 (49.1) | 17 (48.6) | 0.95 |
| Charlson index, median (interquartile range) | 1 (0-3) | 2 (1.3) | 0.17 |
| Charlson index ≥3 | 30 (27.8) | 14 (40.0) | 0.17 |
| Bladder catheter at enrolment | 33 (30.6) | 10 (28.6) | 0.82 |
| Invasive procedure in the urinary tract in previous month | 14 (13.0) | 2 (5.7) | 0.35 |
| Immunosuppressive drugs | 9 (8.3) | 7 (20.0) | 0.06 |
| Present infection |  |  |  |
| Community-acquired infection | 56 (51.9) | 16 (45.7) | 0.52 |
| Healthcare-associated infection | 34 (31.5) | 14 (40.0) | 0.35 |
| Nosocomial infection | 18 (16.7) | 5 (14.3) | 0.73 |
| Flank pain/tenderness | 23 (37.7) | 18 (38.3) | 0.95 |
| Severe sepsis at presentation | 14 (23.0) | 12 (25.5) | 0.75 |
| Early clinical response (day 5-7) | 104 (96.3) | 19 (54.3) | <0.001 |
| Susceptibility of baseline *E. coli* (local laboratory) |  |  |  |
| Amoxicillin-clavulanic acid | 59 (53.7) | 8 (22.9) | 0.001 |
| Cefuroxime axetil | 58 (53.7) | 5 (14.3) | <0.001 |
| Ciprofloxacin | 22 (20.4) | 3 (8.6) | 0.11 |
| Trimethoprim-sulfamethoxazole | 46 (42.6) | 3 (8.6) | 0.11 |
| Mean days until active treatment (SD) | 0.94 (1.14) | 1.11 (1.27) | 0.34 |
| Outcomes |  |  |  |
| Clinical and microbiological cure at test of cure | 86 (79.6) | 19 (54.3) | 0.003 |
| Relapses | 11 (10.1) | 3 (8.5) | 1.0 |
| Reinfection | 7 (6.4) | 1 (2.8) | 0.67 |
| Mortality | 2 (1.8) | 2 (5.7) | 0.24 |

^a^ Includes 13 patients switched to parenteral ertapenem
